# Conference Calendar

**DOI:** 10.1002/cfg.297

**Published:** 2003-06


**Comparative and Functional Genomics**
*Comp Funct Genom* 2003; **4**: 353–355.
Published online in Wiley InterScience (www.interscience.wiley.com). **DOI:** 10.1002/cfg.297

### Conference Calendar

August–November 2003

### Animal models

5th EMBL Mouse Molecular Genetics Meeting, 3–7 Sept

**http://www.embl-heidelberg.de/conferences/MouseGen03/**

17th International Mouse Genome Conference, 9–12 Nov

**http://imgs.org/**

### Bacteria

Molecular Genetics of Bacteria and Phages 2003, 5–10 Aug

**http://www.union.wisc.edu/conference services/phages/**

CSHL — Microbial Pathogenesis and Host Response, 10–14 Sept

**http://meetings.cshl.org/2003/2003host.htm**

FEMS — International Symposium: Functional Genomics of Infectious Diseases and Inflammation, 18–20 Sept

**http://www.fems-microbiology.org/fems/events/design/calendarthis.htm**

### Bioinformatics

5th International Workshop on Information Processing in Cells and Tissues (IPCAT2003), 8–11 Sept

**http://lslwww.epfl.ch/ipcat2003**

3rd Workshop on Algorithms in Bioinformatics, 15–20 Sept

**http://www.conferences.hu/ALGO2003/algo_2003.htm**

CHI — Microarray Data Analysis, 21–23 Sept

**http://www.healthtech.com/2003/mda/index.htm**

CHI — Data Visualization and Interpretation, 24–25 Sept

**http://www.healthtech.com/2003/dvs/index.htm**

5th International Conference on Computational Biology and Genome Informatics, 26–30 Sept

**http://www.cis.uoguelph.ca/~dchiu/cbgi03.htm**

European Conference on Computational Biology (ECCB 2003), 27–30 Sept

**http://www.inra.fr/eccb2003/**

JAX/TIGR — 6th Annual Conference on Computational Genomics, 8–11 Oct

**http://www.jax.org/courses/tigr03.html**

### Evolution/comparative genomics

IMA/RECOMB Satellite Workshop on Comparative Genomics, 20–24 Oct

**http://www.ima.umn.edu/complex/fall/c2.html**

### General genomics

TIGR — 15th International Genome Sequencing and Analysis Conference (GSAC XV), 21–24 Sept

**http://www.tigr.org/conf/gsac/**

ComBio 2003, 28 Sept–2 Oct

**http://www.asbmb.org.au/combio2003/**

Copyright © 2003 John Wiley & Sons, Ltd.

### Human genome

ASHG — 53rd Annual Meeting of ASHG, 4–8 Nov

**http://www.ashg.org/genetics/ashg/menu-annmeet.shtml**

### Pathways and metabolomics

ESF IAFG — Data Integration in Functional Genomics and Proteomics: Biological Pathways, 22–24 Sept

**http://www.functionalgenomics.org.uk/sections/activities/workshops.htm#**

### Pharmacogenomics

IBC — Model Organisms in Drug Discovery, 12–13 Aug

**http://www.lifesciencesinfo.com/2921**

IBC — Biomarkers in Drug Discovery, 12–13 Aug

**http://www.lifesciencesinfo.com/2803**

CSHL — Pharmacogenomics, 24–28 Sept

**http://meetings.cshl.org/hingtonmeetings.htm**

International Conference on Applied Genomics: Translational Genomics in Oncology, 1–4 Oct

**http://www.nddo.org/page_include_istg2003.shtml**

CHI — Genomics on Target, 6–10 Oct

**http://www.genomicsontarget.com/**

ESF IAFG — SNPs Analysis: Tools and Applications, 28–29 Nov

**http://www.functionalgenomics.org.uk/sections/activities/workshops.htm#**

### Plants

2nd Plant-GEMs + 4th GARNet Functional Genomics Meeting, 3–6 Sept

**http://www.york.ac.uk/res/garnet/plantgems2.htm**

ASPB — Plant Genetics 2003: Mechanisms of Genetic Variation, 22–26 Oct

**http://www.aspb.org/meetings/pg-2003/**

### Proteomics

CHI — Protein Biomarkers, 25–26 Aug

**http://www.healthtech.com/2003/bmk/index.htm**

HUPO 2nd Annual World Congress, 8–11 Oct

**http://www.hupo2003.com/**

IBC — Chips to Hits, 27–31 Oct

**http://www.chipstohits.com/**

ACS — Integrating Proteomics into Systems Biology, 9–12 Nov

**http://www.chemistry.org/portal/Chemistry?PID = general.html&DOC = acsprospectives\index.html**

### Structural genomics

EuroConference on Structural Genomics, 5–10 Sept

**http://www.esf.org/esf_euresco_conference.php?language = 0&domain = 2&conference = 14 &meeting = 6**

### Transcriptomics

MGED — 6th International Meeting: MGED6, 3–5 Sept

**http://tagc.univ-mrs.fr/mged6/**

Copyright © 2003 John Wiley & Sons, Ltd.

ESF IAFG — From Gene Array to Gene-a-row, 8–9 Sept

**http://www.functionalgenomics.org.uk/sections/activities/workshops.htm#**

IIR — Bioarrays Europe, 29 Sept–1 Oct

**http://www.bioarrayseurope.com**

IBC — Chips to Hits, 27–31 Oct

**http://www.chipstohits.com/**

### Yeasts and fungi

SGM — 153rd Meeting, Exploiting Genomes: Bases to Megabases in 50 Years, 8–11 Sept

**http://www.sgm.ac.uk/MTGPAGES/Umist.htm**

### Biotech industry

GRC — Global Aspects of Technology Transfer: Biotechnology, 21–26 Sept

**http://www.grc.uri.edu/programs/2003/global.htm**

### Key

ACS, American Chemical Society: **http://www.chemistry.org**
ASHG, American Society for Human Genetics: **http://www.ashg.org**
ASPB, American Society of Plant Biologists: **http://www.aspb.org**
CHI, Cambridge Healthtech Institute: **http://www.healthtech.com**
CSHL, Cold Spring Harbor Laboratories: **http://meetings.cshl.org/index.htm**
EMBL, European Molecular Biology Laboratory: **http://www.embl-heidelberg.de/**
ESF IAFG, European Science Foundation — Integrated Approaches for Functional Genomics: **http://www.functionalgenomics.org.uk/**
FEMS, Federation of European Microbiological Societies: **http://www.fems-microbiology.org/**
GRC, Gordon Research Conferences: **http://www.grc.uri.edu/**
HUPO, Human Proteome Organization: **http://www.hupo.org/**
IBC, IBC Conferences: **http://www.ibc-lifesci.com**
IMA, Institute for Mathematics and Its Applications: **http://www.ima.umn.edu**
JAX, The Jackson Laboratory: **http://www.jax.org**
MGED, Microarray Gene Expression Data society: **http://www.mged.org/**
RECOMB, Conference on Research in Computational Molecular Biology: **http://www.recomb2003.de/recomb.html**
SGM, Society for General Microbiology: **http://www.sgm.ac.uk**
TIGR, The Institute for Genome Research: **http://www.tigr.org**

Copyright © 2003 John Wiley & Sons, Ltd.

